# Tinea capitis due to *Microsporum ferrugineum*: A case of unusual laboratory finding on direct microscopic examination of infected hairs and skin lesions

**DOI:** 10.1016/j.mmcr.2024.100629

**Published:** 2024-02-01

**Authors:** Robab Ebrahimibarogh, Mona Ghazanfari, Tahereh Shokohi, Ensieh Yousefiabdolmaleki, Amir Seyedmousavi, Mohammad T. Hedayati

**Affiliations:** a Student Research Committee Center, Mazandaran University of Medical Sciences, Sari, Iran; b Department of Medical Mycology, School of Medicine, Mazandaran University of Medical Sciences, Sari, Iran; c Invasive Fungi Research Center, Communicable Diseases Institute, Mazandaran University of Medical Sciences, Sari, Iran; d Department of Dermatology, School of Medicine, Mazandaran University of Medical Sciences, Sari, Iran; e Microbiology Service, Department of Laboratory Medicine, Clinical Center, National Institutes of Health, Bethesda, MD, United States

**Keywords:** Tinea captitis, Microsporum ferrugineum, Unusual laboratory finding, Macroconidia

## Abstract

Tinea capitis is a chronic fungal infection of the scalp occurring commonly in children of school age, especially in developing countries. It is caused primarily by the dermatophyte members of genera *Microsporum* and *Trichophyton*. Here we report presence of free-living mycelial stage of dermatophytes, a stage of fungal growth which form in culture medias, around affected hairs and skin scrapings of scalp lesions in a 3-year-old boy presenting with alopecia and multiple scaly, non-erythematous plaques. On direct microscopy examination using 10 % potassium hydroxide, the fungal hyphae and arthrospores were detected in ectothrix form. In addition, we also observed numerous multicellular, thick-walled spindle-shaped macroconidia around hairs and skin scrapings. To our knowledge this is the first study reporting dermatophyte's macroconidia directly seen on clinical samples. Species level identification of the dermatophyte isolate growing on Mycosel™ agar was confirmed by PCR-sequencing of internal transcribed spacer of ribosomal RNA as *Microsporum ferrugineum.* The patient was successfully treated with systemic itraconazole combined with topical ketoconazole shampoo.

## 1 Introduction

Tinea capitis is an important public health concern in developing countries, commonly associated with geographic area under poor hygienic conditions [1]. It can be contracted from another human or an animal or contaminated environment through direct contact. The infection most often presents with pruritic lesions and inflammation that damages both hair and scalp follicles and manifests as erythema, scaling, hair loss, abscesses and/or scar formation after recession of the original infection. Anthropophilic (human) and zoophilic (animal) dermatophytes belong to genera *Trichophyton* and *Microsporum* are the most common causes of tinea capitis, which varies geographically and can change over time [2].

Tinea capitis often should be considered based on a variety of clinical features including scaly patches with alopecia with or without black dots, hair loss with widespread scaling, and severe forms such as kerion and Favus [3]. If tinea capitis is in question, a potassium hydroxide (KOH) preparation or fungal culture should be performed to confirm the diagnosis of tinea. Molecular techniques can be also used to provide presumptive identification of fungal DNA in clinical samples, however it requires to be confirmed by culture as a gold standard diagnostic method. Direct microscopic examination of damaged hairs in KOH often shows fungal elements (arthroconidia or hyphae) within (endothrix infections) and or outside of the hair shaft (ectothrix infections) [4]. To the best of our knowledge, dermatophyte infection of the hair does not present in any other form in direct microscopic examination.

Here, we report for the first-time presence of numerous microconidia, a stage of fungal growth only found in dermatophyte culture medias, around hair shafts and inside skin scrapings of scalp lesion. This is an unusual finding in laboratory diagnosis of tinea capitis using KOH direct microscopic examination of skin scrapings, or hair pluckings from lesions.

## 2 Case

**Fig. 1:**
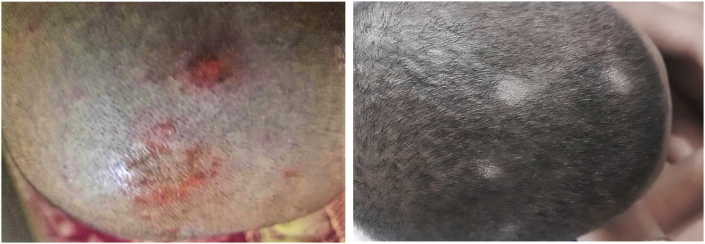
Erythematous scalp lesions and alopecia in the first visit (A). Improved inflammation with continuous hair loss after using topical mupiricin and triamcinolone N.N. for 8 weeks (B).

**Fig. 2:**
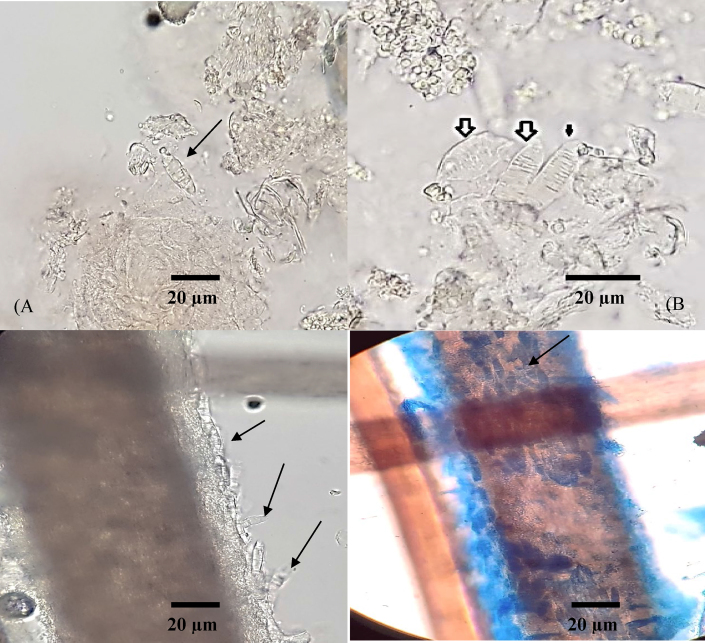
Potassium hydroxide (KOH) preparation of infected scalp lesions and surrounding hair shafts. Several spindle-shaped, multicellular, thick-walled macroconidia inside skin scraping of infected scalp lesions (A and B, arrow heads) and surrounding hair shafts (C and D, arrow heads). Clusters of round (C), basophilic (D) arthrospores embedded within keratin, around hair shafts (ectothrix) and fungal hyphae producing macroconidia (C and D, arrow heads).

A 3-year-old boy was referred to the department of dermatology at Mazandaran University of Medical Sciences, in Sari, Iran in July 2022. Initial physical examination revealed erythematous scalp lesions and alopecia (Fig. 1. A.). Patient received topical mupirocin 2 % ointment and triamcinolone N.N. cream ([Triamcinolone 1mg + Neomycin 2.5mg + Nystatin 100/000U]/g) daily for 8 weeks. Upon follow-up visit, the inflammation was improved, however the hair loss was continued (Fig. 1. B.). He did not have any prior history of other skin microbial infections or animal contact. In depth review of family history revealed that his sibling was a wrestler and had a history of tinea capitis, diagnosed by direct microscopic examination (without culture) and was successfully treated with terbinafine. At this stage, a sample of damaged hairs and scalp lesion was submitted to mycology laboratory for direct microscopy and fungal culture. A KOH preparation was performed and arthroconidia (fungal spores) were present in skin scrapings as well as around the hair shaft. In addition, numerous spindle-shaped, multicellular, thick-walled and echinulate macroconidia were detected (Fig. 2).

**Fig. 3:**
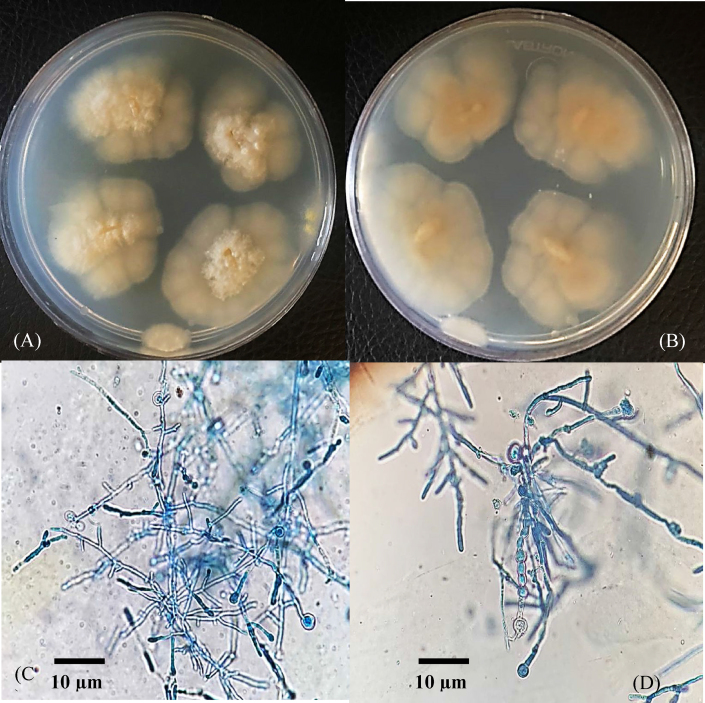
Colony morphology of *Microsporum ferrugineum* on Mycosel™ Agar after 4 weeks' incubation at 30 °C showing glabrous, heaped, wrinkled, sometimes flat, yellow to cream-colored colonies (A), reverse is cream-colored to yellow (B). Conidia absent. Long, narrow hyphae with prominent cross walls bamboo-like hyphae (C), arthroconidia and chlamydospore-like cells (D). (For interpretation of the references to color in this figure legend, the reader is referred to the Web version of this article.)

After 4 weeks incubation at 30 °C, a waxy, glabrous, convoluted thallus with a cream to yellow-colored surface colony furrowed in culture, and orange to yellow color reverse grew on Mycosel™ Agar (Difco, Detroit, MI, USA) culture media, without any obvious pigmentation (Fig. 3. A. and 3. B.). A lactophenol cotton blue wet mount preparation of the fungal colonies, showed large spherical to oval double-walled intercalary-localized chlamydospores, typical “bamboo” shaped hyphae, and racket hyphae lacking micro- and -macroconidia (Fig. 3. C. and 3. D.). The identity of isolate was then confirmed by PCR-sequencing of the internal transcribed spacer (ITS) region of ribosomal DNA (rDNA).

**Fig. 4:**
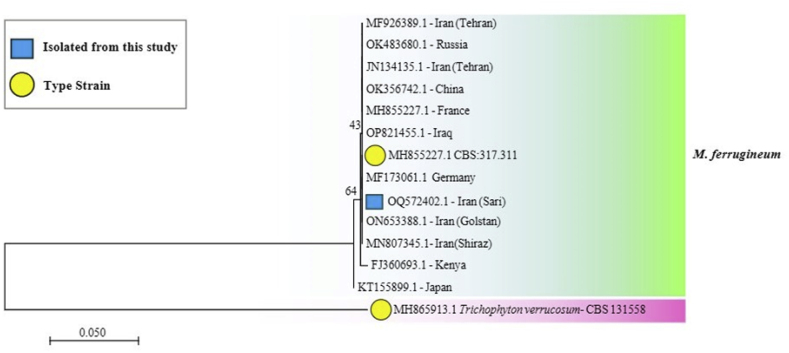
Maximum likelihood phylogenetic tree based on internal transcribed spacer (ITS) region of ribosomal DNA (rDNA), with 1000 bootstrap replications. *Trichophyton verrucosum* was selected as out-group.

The resulting DNA sequences were aligned to both the NCBI Genbank (http://www.ncbi.nlm.nih.gov/genbank) and the International Mycological Association-Westerdijk Fungal Biodiversity Institute (http://www.mycobank.org) databases. Comparison of concatenated ITS sequence (∼500 nucleotides) to both databases yielded sequence identity of 100 % to as *M. ferrugineum* type strain (No. WCH-AV003), which was considered sufficient data to conclude species level identity of this isolate. The corresponding sequences were submitted to Genbank (accession number: OQ572402). A phylogenetic tree including several *M. ferrugineum* isolates from different geographical regions in the world is shown in Fig. 4. The sequencing results were aligned using ClustalW software and phylogenetic analyses were conducted using the MEGA v.11.1 software [5]. A bootstrap analysis was performed with 500 replications. Maximum likelihood then was carried out according to the Kimura two-parameter model.

Antifungal susceptibility testing was conducted in accordance with the Clinical and Laboratory Standards Institute CLSI M38-3rd ed. guidelines [6]. Minimum inhibitory concentrations (μg/ml) were as follows in increasing order: lanoconazole and terbinafineand 0.01 μg/ml, itraconazole and ketoconazole 0.03 μg/ml, griseofulvin 0.5 μg/ml, and fluconazole 8 μg/ml. The patient was successfully treated with ketoconazole shampoo (once a week) and oral itraconazole (5 mg/kg/day) for 4 weeks.

## 3 Discussion

Although tinea capitis primarily occurs in children, an increasing number of cases have also been reported among adults, especially postmenopausal and elderly women [7]. Because culture results may not be available for several weeks, a KOH preparation and direct microscopic examination of damaged hair or skin scraping is a rapid method for confirming the presence of a dermatophyte infection indicating tinea capitis. The procedure can be performed quickly at the point of care. Fungal spores and hyphae can be found within or outside of the hair shaft. In endothrix form, fungal spores (arthroconidia) are found within the hair shaft on direct microscopic examination. This form is predominantly caused by *T. tonsurans* and *T. violaceum* [8]. In ectothrix form, arthroconidia covers outside of the hair shaft. This form is mainly caused by *M. canis*, *M ferrogimnum* and *T. verrucosum*. Favus, or tinea capitis favosa, is a chronic inflammatory dermatophytosis of the scalp, characterized by scutulum formation and scarring atrophy, in which hyphae and air spaces are found within hair shafts. The anthropophilic dermatophyte, *T. schoenleinii* is responsible for over 95 % of favus cases [9].

In the present case, the fungal hyphae and arthrospores were seen on scalp lesions and damaged hairs, which is consistent with ectothrix infection due to *M. ferrogimnum*. Globally, *M. ferrugineum* has been reported from Asia, Eastern Europe and northern Africa, associated with human tinea capitis primarily in children and wrestlers, causing subcutaneous and cutaneous infections, and in a case of deep-seated infection in a patient with CARD9 deficiency [10,11].

Generally, KOH microscopic examination of affected hairs or skin scrapings of tinea capitis lesions due to *Trichophyton* and *Microsporum* may reveal fungal elements such as chains of arthroconidia spores (ectothrix or endothrix) or hyphae. However, in the current case we also observed numerous multicellular, thick-walled spindle-shaped macroconidia around hairs and inside skin scrapings, a stage of fungal growth usually formed in dermatophyte culture medias*,* which is considered an unusual laboratory finding on direct microscopic examination using KOH. Macroconidia-like structures found on the skin or hairs are usually pollen or other species of mold, and dermatophyte macroconidia, which form in culture and or present in soil, are not seen. In agreement with our findings, the spindle shaped macroconidia were also reported under direct microscopic examination of skin scrapings and hair samples from Bali cattles that were clinically suspected to ringworm infection [12]. Presence of free-living mycelial stage of saprophytic fungi, has been also reported in laboratory diagnosis of non-dermatophyte fungal infections. *Aspergillus* conidial heads has been reported as an aid for histopathological diagnosis of angioinvasive pulmonary aspergillosis [13]. *Piedraia hortae* causes a superficial fungal infection known as black piedra, forms nodules on its sexual stage around hair shafts that can be seen in direct microscopic evaluation.

In our study, we anticipate that patient's brother, a wrestler with previous history of tinea capitis, could be the carrier of the *M. ferrugineum* from the contaminated sport environment to this patient. Therefore, as a potential source of infection, presence of macroconidia around hairs and skin scrapings could be a direct transfer from the contaminated environment to patient's head. In addition, macroconidia can be produced inside damaged hairs and scalp lesions under influence of corticosteroids. Triamcinolone is a potent topical corticosteroid that relieves inflammation and itching [14]. Nystatin, a polyene antifungal that damages fungal cell membrane, is used to treat certain kinds of fungal or yeast infections of the skin, however is not effective for dermatophyte infections. In addition, administration of corticosteroid agent may interfere with the therapeutic actions of the antifungal drug and increase fungal growth. This is mainly due to decreased local immunologic host reactions, leading to persistent infection and potential invasion to deeper tissues [15].

The patient was successfully treated with combination of oral itraconazole and topical ketoconazole. Our *in vitro* antifungal susceptibility testing was also consistent with a recently published study indicating that *M. ferrugineum* was sensitive against majority of available antifungal agents currently used in treatment of tinea capitis including clotrimazole, and itraconazole [16].

This case report highlights a novel laboratory finding that macroconidia of *M. ferrugineum,* which usually form in culture, can also appear around infected hairs and inside skin samples alongside fungal arthrospores and hyphae. Further studies are warranted to evaluate the infection biology of this condition among various dermatophyte species.

## Authors contribution

RE and MG did laboratory work, collected the data and drafted the manuscript, TS and EY interpreted the data and revised the manuscript. MTH and AS reviewed the data analysis, interpreted the data, revised the contents and wrote the final version of manuscript. All authors have read and approved the manuscript.

## Ethical Form

Please note that this journal requires full disclosure of all sources of funding and potential conflicts of interest. The journal also requires a declaration that the author(s) have obtained written and signed consent to publish the case report from the patient or legal guardian(s).

The statements on funding, conflict of interest and consent need to be submitted via our Ethical Form that can be downloaded from the submission site www.ees.elsevier.com/mmcr. **Please note that your manuscript will not be considered for publication until the signed Ethical Form has been received.**

## Declaration of competing interest

All other authors declare that there is no conflict of interest.
